# PEPITEM Treatment Ameliorates EAE in Mice by Reducing CNS Inflammation, Leukocyte Infiltration, Demyelination, and Proinflammatory Cytokine Production

**DOI:** 10.3390/ijms242417243

**Published:** 2023-12-08

**Authors:** Mohammed Alassiri, Fahd Al Sufiani, Mohammed Aljohi, Asma Alanazi, Aiman Saud Alhazmi, Bahauddeen M. Alrfaei, Hasan Alnakhli, Yasser A. Alshawakir, Saleh M. Alharby, Abdullah Y. Almubarak, Mohammed Alasseiri, Nora Alorf, Mashan L. Abdullah

**Affiliations:** 1 Department of Basic Sciences, College of Science and Health Professions, King Saud bin Abdulaziz University for Health Sciences (KSAU-HS), King Abdullah International Medical Research Center (KAIMRC), Riyadh 11481, Saudi Arabia; assirim@ksau-hs.edu.sa (M.A.); hazmias@ksau-hs.edu.sa (A.S.A.); 2 Department of Pathology and Laboratory Medicine, King Abdulaziz Medical City (KAMC), Ministry of National Guard-Health Affairs (MNGHA), Riyadh 11481, Saudi Arabia; sufianif@mngha.med.sa (F.A.S.); nikhlih@ngha.med.sa (H.A.); 3 Healthy Aging Research Institute, King Abdulaziz City for Science and Technology (KACST), Riyadh 11442, Saudi Arabia; maljohi@kacst.edu.sa (M.A.); nora.alorf@bristol.ac.uk (N.A.); 4 Department of Basic Medical Sciences, College of Medicine, King Saud bin Abdulaziz University for Health Sciences (KSAU-HS), King Abdullah International Medical Research Center (KAIMRC), King Abdulaziz Medical City (KAMC), Riyadh 11481, Saudi Arabia; anazia@ksau-hs.edu.sa (A.A.); alrfaeiba@mngha.med.sa (B.M.A.); 5 Department of Cellular Therapy and Cancer Research, King Saud bin Abdulaziz University for Health Sciences (KSAU-HS), King Abdullah International Medical Research Center (KAIMRC), Ministry of National Guard-Health Affairs (MNGHA), Riyadh 11481, Saudi Arabia; 6 Department of Experimental Surgery and Animal Laboratory, College of Medicine, King Saud University, Riyadh 12372, Saudi Arabia; yalshawakir@ksu.edu.sa (Y.A.A.); alhsaleh@ksu.edu.sa (S.M.A.); aalmubarak@ksu.edu.sa (A.Y.A.); 7 Department of Medical Laboratory Technology, Faculty of Applied Medical Sciences, University of Tabuk, Tabuk 47512, Saudi Arabia; malasseiri@ut.edu.sa; 8 Department of Experimental Medicine, King Saud bin Abdulaziz University for Health Sciences (KSAU-HS), King Abdullah International Medical Research Center (KAIMRC), Ministry of National Guard-Health Affairs (MNGHA), Riyadh 11481, Saudi Arabia

**Keywords:** multiple sclerosis, EAE model, PEPITEM, demyelination, leukocyte trafficking

## Abstract

To investigate the effect of the therapeutic treatment of the immunopeptide, peptide inhibitor of trans-endothelial migration (PEPITEM) on the severity of disease in a mouse model of experimental autoimmune encephalomyelitis (EAE) as a model for human multiple sclerosis (MS), a series of experiments were conducted. Using C57BL/6 female mice, we dosed the PEPITEM in the EAE model via IP after observing the first sign of inflammation. The disease was induced using MOG35-55 and complete Freund’s adjuvants augmented with pertussis toxin. The EAE score was recorded daily until the end of the experiment (21 days). The histological and immunohistochemistry analysis was conducted on the spinal cord sections. A Western blot analysis was performed to measure the protein concentration of MBP, MAP-2, and N-Cadherin, and ELISA kits were used to measure IL-17 and FOXP3 in the serum and spinal cord lysate. The therapeutic treatment with PEPITEM reduced the CNS infiltration of T cells, and decreased levels of the protein concertations of MBP, MAP-2, and N-Cadherin were observed, in addition to reduced concertations of IL-17 and FOXP3. Using PEPITEM alleviated the severity of the symptoms in the EAE model. Our study revealed the potential of PEPITEM to control inflammation in MS patients and to reduce the harmful effects of synthetic drugs.

## 1. Introduction

Multiple sclerosis (MS) is a chronic autoimmune disorder targeting the central nervous system, including the brain, spinal cord, and optic nerves. This damages the myelin sheath, a protective layer that covers the nerve fibers and supports signal transmission. When the myelin sheath is damaged, the communication between the brain and the rest of the body is impaired [1], resulting in various symptoms and complications, including fatigue, muscle weakness or spasticity, difficulty with coordination and balance, vision problems, cognitive impairment, and mood changes [2].

There are an estimated 2.8 million people with MS globally (35.9 per 100,000 individuals). The prevalence of MS has increased globally, though there are still gaps in the prevalence estimates [3]. The mean age at diagnosis is 32 years, and the combined incidence rate in 75 reporting nations is 2.1 per 100,000 people/year. The prevalence of MS is two times higher in females than in males [4]. It occurs most frequently, with a high prevalence, in North America and Europe (>100/100,000 inhabitants) [5]. The Arabian Gulf regions are in the low-risk zone, based on the Kurtzke classification. However, a recent increase in clinical diagnoses has been noticed (31–55 MS per 100,000 individuals) [6]. Another review published in 2016 confirms this finding [7,8]. In Saudi Arabia, information regarding the impairment and the epidemiological profile of MS patients is lacking. The most current data, however, suggested a high and rising incidence of 40.40/100,000 in the nation [3]. MS can be unpredictable and vary from person to person, ranging from mild to severe disability. There is currently no cure for MS, but there are medications and therapies that assist in symptom management and slowing down their progression [2].

The Peptide Inhibitor of Trans-Endothelial Migration, PEPITEM, is a naturally occurring peptide first identified in 2015 by researchers at the University of Birmingham in the UK [9]. It plays a role in the adiponectin-PEPITEM pathway, which regulates the immune responses and inflammation in the body [9]. More recently, research suggested that PEPITEM may have potential benefits for obesity-related diseases and type 2 diabetes, as it reduces inflammation and improves glucose metabolism [10]. PEPITEM also reduces the recruitment of T cells into the inflamed tissues in peritonitis, hepatic ischemia-reperfusion injury, Salmonella infection, uveitis, and Sjögren’s syndrome [9].

Multiple sclerosis (MS) is a devastating neurological disorder affecting the CNS. The main pathological features of MS are proinflammatory cytokines, demyelination, and secondary axonal degeneration. However, the therapeutic potential of targeting these factors remains unclear. In this study, we aimed to evaluate the effect of PEPITEM, a novel peptide inhibitor of proinflammatory cytokines, on the severity of the disease in the EAE mouse model, which represents many aspects of human MS. EAE is the most commonly used animal model of MS, and most of the current medications have been tested and validated using the EAE model [11]. The similarities between MS and EAE include neuropathology and neurodegeneration in the spinal cord, cerebral cortex, and retina/optic nerve [12]. Furthermore, the mechanism and the pathogenesis of MS have been identified and confirmed in the EAE [13]. However, the model has certain limitations, such as the involvement of B cells, which is well established in MS, whereas the involvement of B cells is not required with the MOG 35–55 EAE model [14,15]. Also, the induction of EAE is induced artificially, not spontaneity like the MS, which makes the model imperfect in studying the etiology of the disease. Moreover, the cortical lesions are well documented in MS but not observed in the EAE model [16,17,18]. Therefore, having a clear and concise research question is very important in choosing a suitable animal model for MS.

We used various methods, such as histological analysis, immunohistochemistry analysis, Western blot analysis, and ELISA assays, to assess the extent and severity of the pathological changes in the different regions of the CNS.

## 2. Results

### 2.1. PEPITEM Reduces Axonal Damage and Demyelination in the Spinal Cord of the EAE Model through Inhibiting the Number of Inflammatory Cells Infiltrates

To investigate the effect of PEPITEM on the induction and the severity of the disease in the EAE model, the mice were given daily injections of PEPITEM or scrambled peptide from the appearance of the first sign of disease until day 21. The mice were weighed and regularly observed using the EAE scoring system and body weight changes.

#### 2.1.1. EAE Scoring Analysis

**Figure 1:**
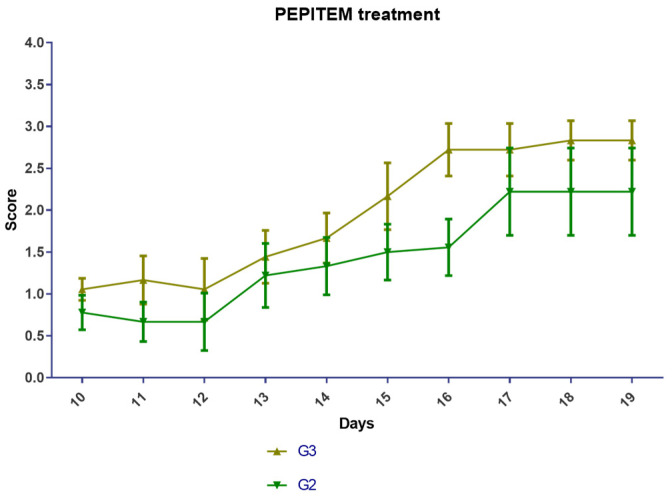
EAE scoring analysis. EAE mice received a daily IP injection of scramble peptide (G2) or PEPITEM (G3) until day 21 post-EAE induction.

Following the Hooke Laboratories induction protocol and EAE recommended scoring system, all the designated groups developed the disease. Typical symptoms developed in the experimental groups (G2 and G3) 10 ± 1 days after the immunization. Both groups were affected in the same period (10 ± 1); the onset symptoms, such as the tip of the tail being limp and weakness in the hind legs, started on day 10 ± 1 after immunization, and the peak symptoms, such as complete hind leg and partial front leg paralysis and death were observed on day 17 (Figure 1 and Table 1). The mice scoring in G2 and G3 were equally likely to score high or low.

#### 2.1.2. Changes in Body Weights of EAE Mice

**Figure 2:**
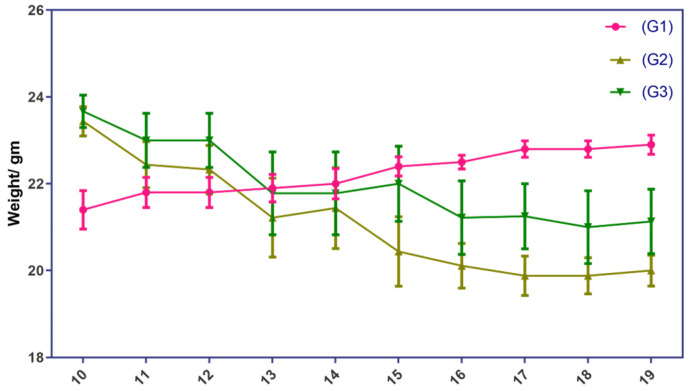
Changes in body weights of EAE mice G1 group represents the normal control group, whereas G2 and G3 represent the EAE mice groups. EAE mice received a daily IP injection of scramble peptide (G2) or PEPITEM (G3) until day 21 post-EAE induction.

The body weight of the EAE mice showed a slight downward trend over the course of the experiment, but the changes were not statistically significant. The induction of the disease and the administration of PEPITEM did not affect the mice’s body weights in a noticeable way. This may be attributed to the variable response of the EAE mouse model within the same group. Some animals exhibited greater body weight loss due to severe hind limb paralysis, while others had mild symptoms (Figure 2). Although the difference was not statistically significant, PEPETIM appeared to rescue the weight of the mice. The weight of the mice in group G3 was closer to the weight of the mice in group G1 than the weight of the mice in group G2.

#### 2.1.3. Histopathological Analysis

**Figure 3:**
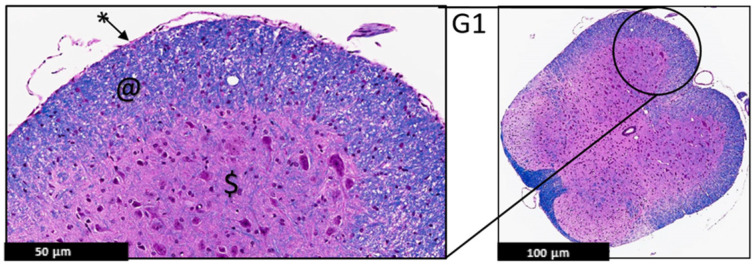
Representative histology of the spinal cords in EAE mice. G1: Cross-section from spinal cord. Panoramic view of normal spinal cord. Intact thin leptomeninges (*), well demarcation between white (@) and gray ($) matter, uniformly myelinated white matter (H&E/LFB stain), (*n* = 3).

**Figure 4:**
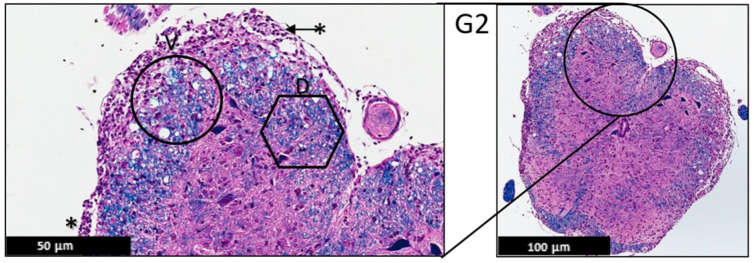
Representative histology of the spinal cords in EAE mice. G2: Cross-section from spinal cord staining. EAE mice received a daily IP injection of scramble peptide (G2) until day 21 post-EAE induction. Leptomeninges (*) are thickened by lymphocytic infiltrates, which appear to extend to the adjacent white matter. There is bilateral patchy demyelination (D) in white matter with vacuolization (V) (H&E/LFB stain), (*n* = 3).

**Figure 5:**
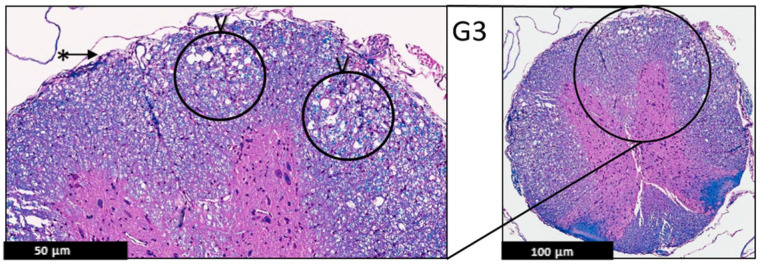
Representative histology of the spinal cords in EAE mice. G3: Cross-section from spinal cord staining. EAE mice received a daily IP injection PEPITEM (G3) until day 21 post-EAE induction. There is only patchy vacuolization (V) in white matter with no evidence of demyelination (uniformly myelinated white matter). The leptomeninges (*) appear thin with no evidence of inflammation (H&E/LFB stain), (*n* = 3).

The histopathological changes were evaluated by a certified neuropathologist using the spinal cord of the EAE mice stained with H&E and LFB under the light microscope. This analysis focused on the demarcation between white and gray matter, the presence or absence of demyelination, leptomeninges appearance, and the presence of any sign of inflammation in the leptomeninges or the CNS parenchyma, such as Lymphocytic infiltrate. This analysis showed a protective effect of PEPITEM on the treatment group (G3), evident by uniformly myelinated white matter and no evidence of demyelination. The leptomeninges appeared thin with no evidence of inflammatory cells, compared to the control group (G2) (Figure 3, Figure 4 and Figure 5).

**Figure 6:**
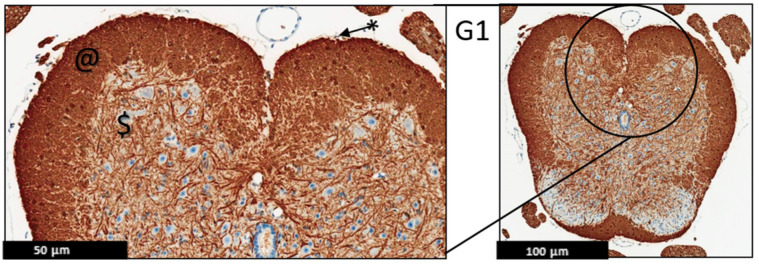
Representative histology of the spinal cords in EAE mice. G1: Cross-section from MBP immunohistochemistry spinal cord staining. Panoramic view of normal spinal cord stained with myelin basic protein. Again, there is well demarcation between white (@) and gray ($) matter; intact thin leptomeninges (*). The staining is uniform in white matter, (*n* = 3).

**Figure 7:**
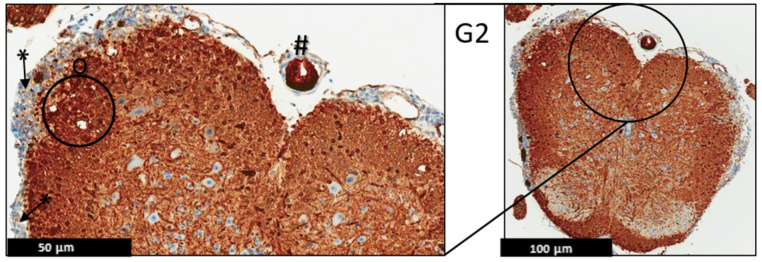
Representative histology of the spinal cords in EAE mice. G2: Cross-section from MBP immunohistochemistry spinal cord staining. EAE mice received a daily IP injection of scramble peptide (G2) until day 21 post-EAE induction. Leptomeninges (*) are thickened by lymphocytic infiltrates, which appear to extend to the adjacent white matter. There is no significant loss of myelin basic protein staining in white matter (properly heavy staining—note, there is non-specific staining in endothelial cells of some blood vessels (#)). Patchy vacuolation (O) is evident, (*n* = 3).

**Figure 8:**
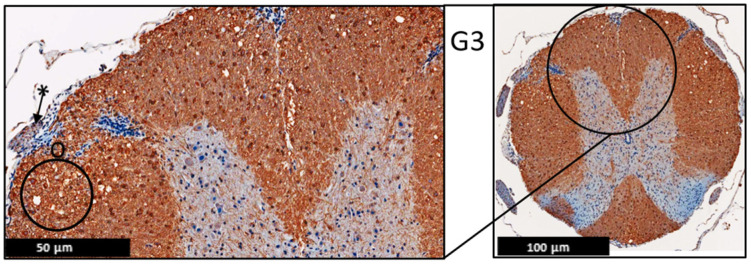
Representative histology of the spinal cords in EAE mice. G3: Cross-section from MBP immunohistochemistry spinal cord staining. EAE mice received a daily IP injection of PEPITEM (G3) until day 21 post-EAE induction. There is only patchy vacuolization (O) in white matter with no evidence of demyelination (uniformly myelinated white matter). The leptomeninges (*) appear thin with minimal inflammation. Differential spine section from the prior H&E/LFP staining, (*n* = 3).

**Figure 9:**
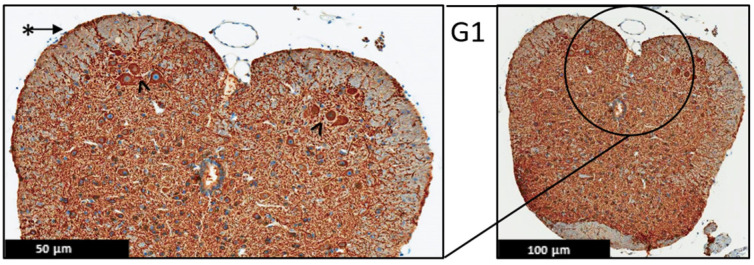
Representative histology of the spinal cords in EAE mice. G1: Cross-section from MAP2 immunohistochemistry spinal cord staining. Panoramic view of normal spinal cord. Intact thin leptomeninges (*). The stain highlights the neurons in the anterior horn with intact meshwork (^), (*n* = 3).

**Figure 10:**
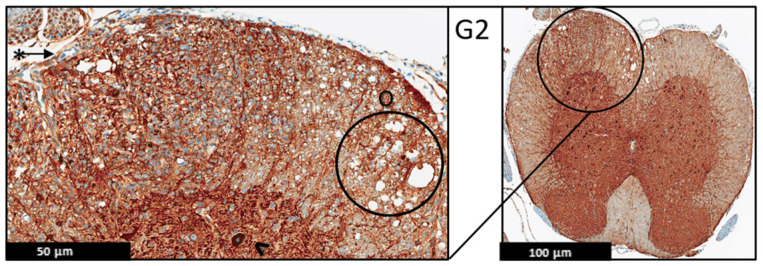
Representative histology of the spinal cords in EAE mice. G2: Cross-section from MAP2 immunohistochemistry spinal cord staining. EAE mice received a daily IP injection of scramble peptide (G2) until day 21 post-EAE induction. There is patchy vacuolation (O) in the white matter with increased cellularity of lymphocytes in leptomeninges (*). The neurons of anterior horn are present and diffusely stained (^), (*n* = 3).

**Figure 11:**
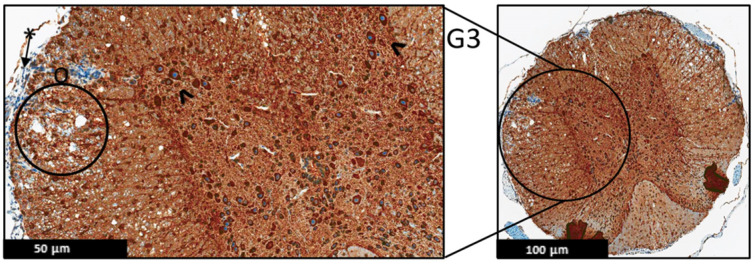
Representative histology of the spinal cords in EAE mice. G3: Cross-section from MAP2 Immunohistochemistry spinal cord staining. EAE mice received a daily IP injection of PEPITEM (G3) until day 21 post-EAE induction. There is patchy vacuolation (O) in the white matter. There is no significant lymphocytic inflammation in leptomeninges (*). The neurons of anterior horn are present and diffusely stained (^), (*n* = 3).

Moreover, the immunohistochemistry technique was used to evaluate the protein expression of the MBP and MAP-2 in the spinal cord of the EAE mice (Figure 6, Figure 7, Figure 8, Figure 9, Figure 10 and Figure 11), and the results showed that there were no significant changes in the protein expression of MBP and MAP-2 in both groups; however, inflammatory cells were observed in the leptomeninges of G2 only. Patchy vacuolation was observed in both groups (Figure 6, Figure 7, Figure 8, Figure 9, Figure 10 and Figure 11).

#### 2.1.4. Protein Expression of MBP, MAP-2, and N Cadherin

**Figure 12:**
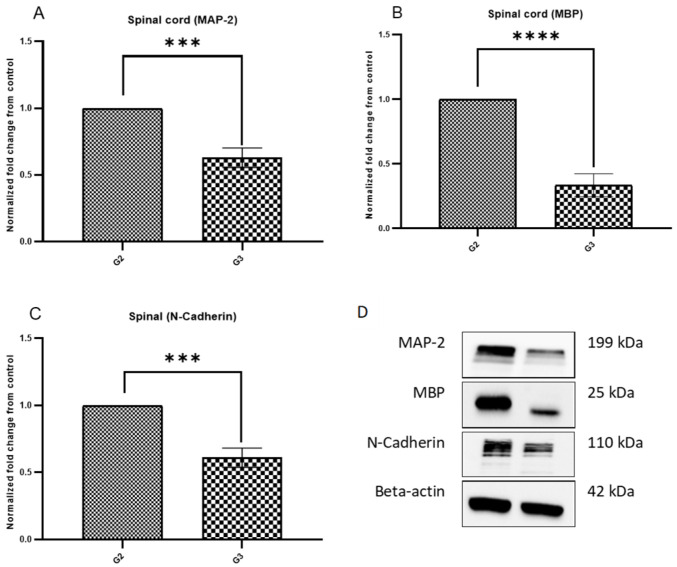
MBP, MAP-2, and N-Cadherin protein expression in the spinal cord of EAE mice on day 21. EAE mice received a daily IP injection of scramble peptide (G2) or PEPITEM (G3) until day 21 post-EAE induction. (**A**–**D**) showed the protein expression in G2 and G3 groups. The protein expression was evaluated by Western blot. Mean ± SEM is depicted (*n* = 4 per group in triplicate). Note: *** *p* < 0.0002, **** *p* < 0.0001.

The Western blot (WB) technique was used to evaluate the protein expression of MBP, MAP-2, and N-Cadherin on the spinal cord of the mice on day 21. The data showed a statistically significant decrease in the protein expression of MBP, MAP-2, and N-Cadherin, indicating fewer inflammatory changes in the PEPITEM-treated group G3 than in G2 (Figure 12).

### 2.2. PEPITEM Has an Anti-Inflammatory Effect in the Spinal Cord of the EAE Model by Controlling the Levels of IL-17 and FOXP3 in the Spinal Cord Lysate

**Figure 13:**
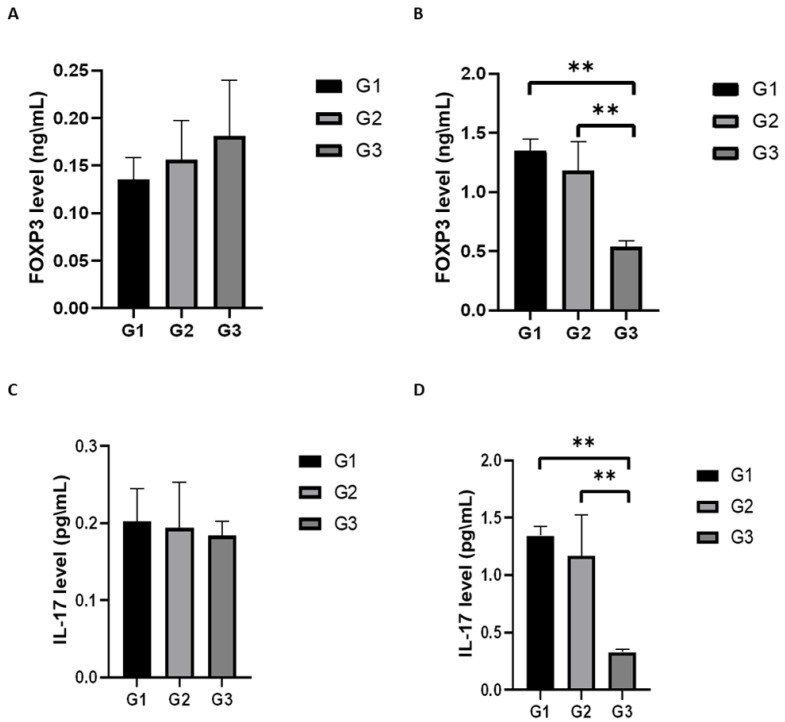
Quantitative measurement of FOXP3 and IL 17 levels in EAE mice serum and spinal cord lysate. EAE mice received a daily IP injection of scramble peptide (G2) or PEPITEM (G3) until day 21 post-EAE induction. The level of FOXP3 in serum and spinal cord lysate (**A**,**B**) and the level of IL 17 in serum and spinal cord lysate (**C**,**D**) were assessed using ELISA commercial kits. Data are expressed as Mean ± SEM (*n* = 3 per group in duplicate). Note: ** *p* < 0.01.

ELISA kits for mouse IL-17 and FOXP3 were used for the quantitative measurement of both proteins in the serum and spinal cord lysate. The data indicated that the circulating levels of IL-17 and FOXP3 for all three groups were in the same range; no statistical difference was observed. However, there was a statistical difference in the levels of IL-17 and FOXP3 in the spinal cord lysate. PEPITEM significantly decreased the levels of IL-17 and FOXP3 in the spinal cord lysate of the EAE mice (Figure 13).

## 3. Discussion

The aim of this study was to investigate the anti-inflammatory effect of PEPITEM on the local inflammation of the white matter in the CNS, the main feature of MS. We used the EAE mouse model of MS to evaluate the impact of PEPITEM on the inflammatory infiltrates and the demyelination in the CNS. Our results provide compelling evidence that PEPITEM is a potent anti-inflammatory and neuroprotective agent that can significantly reduce the number and severity of inflammatory lesions in the CNS while preserving myelin integrity. These findings suggest that PEPITEM has the potential to be a novel therapeutic agent for the treatment of multiple sclerosis and other demyelinating diseases.

EAE is a widely used animal model of MS that mimics the human disease’s inflammatory and demyelinating processes. One of the methods to assess the disease severity in EAE is to measure the clinical signs of paralysis in the animals, namely, EAE scoring. However, the subjectivity of the evaluator is a potential limitation of this method. This was mitigated by blinding the evaluators to the treatment group of this study. In this study, we compared the effects of PEPITEM, a novel peptide-based immunotherapy, on the EAE scoring of mice. We found that the PEPITEM-treated mice (G3) showed fewer symptoms of paralysis compared to the G2 mice, which was not statistically significant (Figure 1). This suggests that PEPITEM with the 100 mg/mL IP may have protective effects on the nervous system, but it is insufficient to reverse the disease progression symptoms or mice scoring. Another explanation is the stress caused by the daily IP injections. It is established that stress exacerbates the signs used for scoring, which results in misleading scores [19]. The same observation was observed in body weight. Both experimental groups (G2 and G3) lost weight compared to the healthy group (G1), which was expected due to the burden of the disease and the stress caused by the daily IP injections [20].

The histological analysis of pathological changes on the H&E and LFB stained sections of the spinal cord with EAE revealed that the PEPITEM limited the number of inflammatory filtrates and, as a result, reduced demyelination in the white matter in the spinal cord.

These data are also evident in the light microscopy analysis, which indicated less inflammation, less demyelination, and fewer leukocyte infiltrates to the leptomeninges in G3 compared to G2. The PEPITEM treatment had an anti-inflammatory effect in the EAE model and improved the symptoms. The findings of this study are similar to a recent study by Pezhman et al. The authors examined the effect of PEPITEM on leukocyte trafficking in a high-fed obesogenic diet and concluded that PEPITEM reduced the inflammation caused by obesity via restraining leukocyte trafficking [10]. Another study reported that PEPITEM inhibited T-cell trafficking and glomerulonephritis in a mouse model of SLE and preserved the structure of the kidney [21]. PEPITEM reduced the recruitment of T cells into the inflamed tissues in peritonitis, hepatic ischemia-reperfusion injury, Salmonella infection, uveitis, and Sjögren’s syndrome [9]. PEPITEM is a novel peptide discovered in 2015, and its mechanism of action is poorly understood. However, a recent study reported exciting data about the indirect involvement of the sphingosine-1-phosphate (S1P) [9,22]. Chung et al. reported that during demyelination, very-long-chain fatty acids (VLCFAs) are released from the myelin and exposed to glial cells, converting them into sphingosine-1-phosphate (S1P), which leads to neuroinflammation and activates the NF-κB pathway [22]. Interestingly, when PEPITEM binds to the endothelial receptor cadherin-15, it stimulates the synthesis and the release of S1P and subsequently inhibits T-cell trafficking by binding to its receptors SIPR1 and SIPR4. Investigating the levels of VLCFAS in our model will be interesting and important to understanding the molecular pathways of the protective effect of PEPITEM.

In addition to the histological analysis, the protein expression of MBP and MAP-2 in the spinal cord of the mice was assessed using immunohistochemistry techniques. There were no significant changes in the protein expression of MBP and MAP-2 in the experimental groups G2 or G3. To validate these results with a more accurate technique, we used WB because sometimes the formalin-fixed, paraffin-embedded (FFPE) section may lead to the destruction of some antigens, which makes immunohistochemistry challenging. The WB analysis revealed that the levels of MBP and MAP-2 in the PEPITEM-treated group G3 were lower compared to G2, supporting the anti-inflammatory effect of PEPITEM on the white matter of the CNS. Collectively, we used different techniques to understand these proteins’ expressions better. Our findings suggest that PEPITEM could be helpful in reducing inflammation in the central nervous system. This could have important implications for future treatments. It is known that CNS degenerative diseases or damage lead to the release of proteins in the neurons and oligodendroglia [23]. The low level of MPB in the G3 group indicated less CNS damage or degenerative changes. Clinically, increased levels of MBP are associated with demyelination in infants with hydrocephalus [23]. The literature suggests that the EAE model shows no reduction in the MPB with extensive demyelination, which supports the hypothesis that the reduction in MBP in G3 is caused by the administration of PEPITEM.

Microtubules (MTs) play a significant role in neuronal development and generation as they provide physical support to the neuronal processes and are an indicator of the neuron’s integrity and polarity [24]. Disorganization between the MTs and MAP-2 is linked to many neurodegenerative diseases [25,26,27]. The data from the WB analysis of MAP-2 indicated low levels of MAP-2 in the PEPITEM-treated group G3, compared to G2, which is in accordance with the WB data of MBP. In MS patients, the literature reported that MAP-2 expressing exon 13 (MAP-2+13) is expressed in oligodendrocytes associated with demyelinated lesions [28]. The low levels of MAP-2 in the PEPITEM-treated group could be attributed to its anti-inflammatory effect, resulting in less neural damage and a lower demand for MAP-2. 

Similar to MAP-2, the WB analysis of the N-cadherin indicated low levels in the PEPITEM-treated group G3 compared to G2. N-cadherin is a cell–cell adhesion molecule that supports neuronal structure and differentiation [29]. The N-cadherin data are consistent with the MAP-2 data due to the PEPITEM limiting leukocyte trafficking and reducing demyelination. Since there is less neuronal damage, there is no demand for activating the neuronal machinery to generate more N-cadherin and MAP-2. Interestingly, cancer research suggests that N-cadherin in cancerous cells, in addition to other molecules, enables tumor invasion [30]. The mortality rate of patients with ovarian cancer is increased when N-cadherin is overexpressed [31].

One of the hallmarks of EAE is the local inflammation in the CNS, which is mediated by a variety of proinflammatory cytokines, one of which is IL-17. The literature suggests that IL-17 and IL-17-producing cells play a key role in mediating many of the autoimmune diseases, such as MS [32]. FOXP3 is an important transcription factor of regulatory T cells and is essential for the functions of the regulatory T cells [33]. We assessed the circulating levels of IL-17 and FOXP3 in the serum and spinal cord lysate and found that the circulating levels of IL-17 and FOXP3 in the serum for all the groups (G1, G2, G3) were in the same range with no statistical difference. However, the circulating levels of IL-17 and FOXP3 in spinal cord lysate were significantly reduced in the PEPITEM-treated group compared to the G2 and G1. This finding is in parallel with the histological and WB analysis as we observed fewer inflammatory infiltrates and less demyelination attributed to the inhibition of leukocyte trafficking by the anti-inflammatory effect of PEPITEM. It is the general consensus that the reduction in Foxp3 is associated with a proinflammatory effect; nevertheless, recent findings challenge this consensus. Zhou et al. demonstrated that the decrease in FoxP3+Tregs inhibits cancer proliferation and tumor growth [34]. Moreover, another study suggests that the expression of FoxP3+ does not necessarily demonstrate immune suppressive activity [35]. Hence, the decrease in Foxp3 in the spinal cord of the EAE model could be one of the underlying mechanisms by which PEPITEM exerts its anti-inflammatory effect. The fact that the proinflammatory cytokine levels were reduced in the spinal cord indicates the downregulation of the inflammatory reactions, the suppression of chemotactic signal, and, subsequently, the inhibition of leukocyte recruitment to the inflamed tissues. Clinically, when a patient is diagnosed with MS, the target of treatment initiation is to reduce CNS inflammation [36]. The literature supports this conclusion through the work of McGinley et al., who developed the IL-17 A^−/−^ mice and reported that mice deficient in IL-17 are resistant to developing EAE after induction [37]. PEPITEM is an excellent candidate for drug therapy studies seeking to control the inflammatory response in MS patients.

A possible direction for future studies is to increase the sample size to minimize the impact of animal variation on the results. This would enhance the statistical power and reliability of the findings. Another suggestion is to investigate different doses and routes of PEPITEM administration to optimize its efficacy and safety. Finally, it would be interesting to confirm the effect of PEPITEM on inflammation by measuring more inflammatory markers and cytokines in the serum and tissues of the treated animals.

The result of this study cannot be extrapolated to other concentrations or modes of injection. Further research is needed to evaluate the efficacy and safety of different dosages and delivery methods. These data also highlight one of the limitations of EAE scoring as a measure of disease severity, as it may not capture the subtle changes in the pathology or function of the central nervous system. Moreover, the subjectivity of the evaluator during mice scoring is a potential limitation of this module.

## 4. Materials and Methods

### 4.1. Animals

Hooke Laboratories provided immunization peptides and toxins to develop EAE in the mice model. C57BL/6 female mice were used from the animal facility at the Experimental Surgery Center at King Saud University (KSU). Three experimental groups of mice were randomly assigned, and each group consisted of 10 female mice aged 9–13 weeks (21 ± 3 gm). The mice were group-housed in standard laboratory mice cages with bedding and nesting material. The animal facility was maintained at 22 ± 2 °C and 50 ± 10% relative humidity. The mice were subjected to a 12-h light and a 12-h dark cycle, with lights on at 7:00 a.m. and off at 7:00 p.m. The mice had free access to water, water gel on the cage floor, and food. They were weighed and evaluated daily by the veterinarians. The experimental design was approved by the Institutional Animal Care and Use Committee (IACUC) at King Abdullah International Medical Research Center (KAIMRC) (RC-19/084/R), and it was approved by the Research Ethics Committee at King Saud University (KSU) (KSU-SE-19-10).

### 4.2. Experimental Design

Three experimental groups of mice were randomly assigned to the normal control group (G1), the EAE-induced group receiving the scrambled peptide (G2), and the EAE-induced group receiving PEPITEM (G3). Any mice from G2 or G3 who did not develop the EAE signs after 10 ± 1 days from the start of the experiment were excluded from this study and euthanized.

### 4.3. Reagents

EAE induction kits were purchased from Hooke Laboratories, which contained pre-filled syringes filled with myelin oligodendrocyte glycoprotein 35–55 (MOG35-55/mL) emulsion with complete Freund’s adjuvant and vials containing pertussis toxin (PTX) in glycerol buffer (Hooke Laboratories, LLC, Lawrence, MA, USA). The PEPITEM and scrambled peptide were synthesized by Thermo Fisher Scientific (Waltham, MA, USA). Recombinant anti-myelin basic proteins antibody (MBP), microtubule-associated protein-2 (MAP-2) antibody, Recombinant Anti-N Cadherin antibody, Recombinant Anti-GAPDH Cadherin antibody, and Quant-TI protein assays kit were purchased from Abcam (Cambridge, UK). The acrylamide/bis solution and thick blot filter paper were purchased from BioRad (Bio-Rad Laboratories, Richmond, CA, USA). The Mouse Interleukin 17 (IL17) and Mouse Forkhead Box Protein P3 (FOXP3) ELISA kits (Cat no: MBS455642 and MBS452652) were purchased from MyBioSource, Inc. (San Diego, CA, USA).

### 4.4. Induction of EAE Animal Model

To induce the disease, the EAE kits were used from Hooke Laboratories. The kits were freshly prepared, used within the expiration date, and kept in the refrigerator (2–8 degrees). Following the manufacturer’s instructions, the mice were injected with the immunization with an emulsion of MOG35-55 in complete Freund’s adjuvant (CFA). Two injections of pertussis toxin in PBS were administered to all mice on day 0 and day 1. Briefly, using the mouse restraint cage, the antigen emulsion was subcutaneously administrated at the upper and lower back’s midline at a ratio of 0.1 mL/site (0.2 mL/mouse total). After two hours, the PTX toxin was freshly prepared and diluted following the manufacturer’s instructions and administrated intraperitoneal (IP) at 0.1 mL/dose on day 0 and day 1. The G1 mice were given normal saline injections.

### 4.5. PEPITEM Treatment

Since this study was therapeutic, the daily injections started on day 10 ± 1 post-induction, when the first sign of inflammation appeared until the end of the experiment on day 21. Daily PEPITEM IP injections (concentration of 100 mg/mL; 200 µL total volume per injection) were given according to our previous work [9].

### 4.6. Animal Observations

The mice were observed daily in terms of weight, EAE score, behavior, incidents, and mortality. The EAE scoring system was adapted from the Hooke Laboratories induction kit manual (https://hookelabs.com/services/cro/eae/MouseEAEscoring.html, accessed on 10 February 2021), and the scores were recorded daily by the animal caretakers and the research team. A 0 score indicated no symptoms, 0.5: limp in the tip of the tail, 1: complete tail flaccid, 1.5: tail flaccid plus walking imbalance, 2: tail flaccid plus hind legs inhibition, 2.5: tail flaccid and hind legs weakness, 3: complete tail lip and hind legs paralysis, 3.5: additional to complete tail lip and hind legs paralysis, when a mouse cannot return to a normal position when put on its side. 4: complete tail lip and hind legs paralysis and front limbs partial inhibition, and 5: severe complete paralysis, or mouse found dead, euthanasia was recommended.

### 4.7. Tissues Collection

Humane euthanasia was conducted by using an overdose of anesthesia. Since this study focused on the acute stage of the disease, the EAE mice were sacrificed on day 22, and half of the mice were fixed. Briefly, half of the mice were euthanized and perfused with cold PBS and paraformaldehyde through the left ventricles. The organs were harvested, fixed, and embedded in paraffin for Hematoxylin and eosin stain (H&E), Luxol fast blue (LFB), and immunohistochemistry (IHC) staining. The remaining half of the mice were sacrificed, and the organs were harvested quickly and immediately frozen in liquid nitrogen and kept at −80 °C for the Western blot (WB) detection.

### 4.8. Histopathological Analysis

The lumbar region of the spinal cord was the target of this analysis for two reasons. Firstly, the lumbar region was affected quickly and frequently in this model. Secondly, the motor neurons that innervate the hind limb’s muscle reside in the lumbar region. Therefore, 4 µm thickness sections from the spinal cord were sectioned from paraffin blocks using a microtome. The sections were stained with H&E and LFB and immersed in xylene for dewaxing. The sections were dehydrated by immersion in a gradient of alcohol for five minutes and stained with Harris hematoxylin for 1 min, 10 min for eosin, and Luxol fast blue. Finally, the sections were dehydrated with alcohol and mounted for light microscopy using the ZEISS Axio Imager 2 microscope from Carl Zeiss GmbH (München, Germany) and Aperio ScanScope AT slide scanner from Leica Biosystems (Deer Park, IL, USA).

### 4.9. Immunohistochemistry

Automated immunohistochemistry was performed using the Ventana benchmark system following the manufacturer’s instructions. Briefly, 10% neutral buffered formalin-fixed tissue slides were used and incubated with the primary antibodies of MBP and MAP-2, followed by incubation with a secondary antibody combined with a chromogenic substrate.

### 4.10. Spinal Cord Homogenate Preparation

The spinal cord samples were weighed and homogenized using a plastic grinding rod in ice-cold M-PER™ mammalian protein extraction reagent (Catalog No 78501) from Thermo Scientific (Waltham, MA, USA) at a concentration of 10 µL/µg tissue and centrifuged at 14,000 rpm and 4 °C for 15 min. The protein concentration of the supernatant for each sample was measured according to the manufacturer’s instructions using Qubit™ protein assay kits (Catalog No Q33211) from Thermo Fisher Scientific (Waltham, MA, USA).

### 4.11. Western Blot Analysis

One µg of the mouse spinal cord homogenate was separated by 10% sodium dodecyl sulfate-polyacrylamide gel electrophoresis (SDS-PAGE) and transferred to a PVDF blotting membrane (Catalog No 10600021) from GE Healthcare (Freiburg, Germany), blocked with 5% skimmed dried milk for 60 min. Then incubated with primary anti-beta Actin antibody (1:2000) (Catalog No ab6276), anti-MAP-2 (1:1000) antibody (Catalog No ab32454), MBP antibody (1:1000) (Catalog No ab216668), and anti-N cadherin (1:1000) (Catalog No ab76011) in blocking solution at 4 °C overnight; all the antibodies were from Abcam (Cambridge, United Kingdom). After washing three times (5 min each), the membranes were incubated with goat anti-rabbit (Catalog No ab205718) or goat anti-mouse (Catalog No ab205719) (1:10,000) from Abcam (Cambridge, United Kingdom). After washing four times (5 min each), it was incubated in an ECL Western blotting substrate kit (Catalog No ab65623) from Abcam (Cambridge, United Kingdom) for 2 min. Each band on the membranes was detected using a ChemiDoc Touch imaging system from Bio-Rad (Hercules, CA, USA). The data were analyzed semi-quantitatively using the Image Lab software v6.1.0.7.

### 4.12. Quantification of the Serum and Spinal Cord Levels of FOXP3 and IL-17

The quantification of the serum and spinal cord levels of FOXP3 and IL-17 was measured with ELISA kits (MyBioSource, Inc. (San Diego, CA, USA), following the manufacturer’s instructions. Briefly, the microtiter plate was pre-coated with IL-17 or FOXP3, and standards or samples (serum or spinal cord homogenate) were added to the designed wells, which were treated with a biotin-conjugated antibody specific for IL-17 or FOXP3. After that, horseradish peroxidase (HRP), conjugated with avidin, was added to each well and left for incubation. The wells containing IL-17 or FOXP3 exhibited changes in color, which were measured spectrophotometrically at a wavelength of 450 nm. Finally, the concentrations of the IL-17 or FOXP3 were calculated using the optical density of the samples to the standard curve.

### 4.13. Statistical Analysis

The results from the quantitative experiments were expressed as mean ± SEM. The data were analyzed using the GraphPad Prism software v8.0.2 (GraphPad Prism 8 software Inc., La Jolla, CA, USA). To compare the mean values of three or more groups, we used a one-way analysis of variance (ANOVA) test, followed by the Tuki Kramer multiple comparisons test as a post hoc test. The significance level of *p* < 0.05 was used to determine whether the results were statistically significant.

## 5. Conclusions

The therapeutic treatment with PEPITEM reduced the CNS infiltration of T cells by modulating leukocyte trafficking into the perivascular space of the mice’s spinal cord. In addition, decreased levels of the protein concertation of MBP, MAP-2, and N-Cadherin suggested less axonal damage and subsequently decreased demyelination. The reduced concertation of the proinflammatory cytokines IL-17 and FOXP3 suggests a suppression of the inflammation in the CNS. Collectively, the therapeutic treatment of PEPITEM alleviated the severity of the symptoms in the EAE model. Our study revealed the potential of PEPITEM to control inflammation in MS patients and to reduce the harmful effects of synthetic drugs. PEPITEM offers a novel and safe strategy for drug therapy in MS, opening new avenues for research and treatment.

## Figures and Tables

**Table 1:** Experimental design.

	G1	G2	G3
Starting number of mice	10	10	10
Mice that developed EAE after (10 ± 1) days	0	10	10
Mice excluded due to death or reached a score of 5	0	1	1
The final number of mice included in the statistic	10	9	9

## Data Availability

The data presented in this study are available on a reasonable request from the corresponding author.
